# The relationship between in-hospital mortality and Naples prognostic score in patients undergoing tricuspid valve surgery

**DOI:** 10.1590/1806-9282.20250931

**Published:** 2026-06-15

**Authors:** Samet Sevinc, Yusuf Demir, Seda Tükenmez Karakurt, Hüseyin Karakurt, Salih Guler

**Affiliations:** 1 Mehmet Akif Ersoy Thoracic and Cardiovascular Surgery Training and Research Hospital, Department of Cardiology – Istanbul, Turkey.; 2 Bakircay University, Cigli Training and Research Hospital – İzmir, Turkey.; 3 Mehmet Akif Ersoy Thoracic and Cardiovascular Surgery Training and Research Hospital, Department of Cardiovascular Surgery – Istanbul, Turkey.

**Keywords:** Mortality, Inflammation, Malnutrition

## Abstract

**OBJECTIVE:**

Predicting in-hospital mortality rates following tricuspid valve surgery is imperative, especially in light of the recent advancements in treatment modalities. The Naples prognostic score serves as an indicator of systemic inflammation, malnutrition, and prognostic outcomes across a spectrum of medical conditions. In this investigation, we sought to assess the efficacy of the Naples prognostic score in predicting in-hospital mortality among patients undergoing tricuspid valve surgery.

**METHODS:**

A total of 360 consecutive patients diagnosed with severe tricuspid valve disease, who underwent tricuspid valve surgery, were included in this retrospective study. The study population was categorized based on the Naples prognostic score classification into two groups: low (0–1–2) and high (3–4).

**RESULTS:**

The study's primary outcome—in-hospital mortality—was significantly higher in patients with a high Naples prognostic score group than in those with a low Naples prognostic score group (35 [28.2%] vs. 8 [5.1%], p<0.001). In the receiver operating characteristic analysis, the Naples prognostic score optimal cut-off value of >2 predicted in-hospital mortality with 81.4% sensitivity and 62.5% specificity (area under the curve: 0.782 [95%CI 0.729–0.829, p<0.0001]).

**CONCLUSION:**

The findings of this study indicate that the Naples prognostic score serves as an independent predictor of in-hospital mortality among patients undergoing tricuspid valve surgery.

## INTRODUCTION

Tricuspid valve (TV) disease, particularly tricuspid regurgitation (TR), has garnered substantially less attention in comparison to aortic and mitral valve diseases, despite the frequency of TR's occurrence. This condition is frequently designated as the "forgotten valve"^1,2^. Despite the recommendations outlined in the guidelines, the surgical repair of the TV continues to be underutilized^3^. It has been reported that the in-hospital mortality rate for surgically treated TV diseases is 8.7%^4^. Presently, the logistic EuroSCORE, EuroSCORE II, and the Society of Thoracic Surgeons (STS) cardiac surgery risk model are not specifically tailored to forecast outcomes for these infrequent procedures. Although there have been some initiatives to develop a risk assessment model explicitly for tricuspid valve surgery (TVS), progress remains limited^5^.

The Naples prognostic score (NPS) constitutes a significant scoring system that incorporates serum albumin, total cholesterol level, the neutrophil-lymphocyte ratio (NLR), and the lymphocyte-monocyte ratio (LMR). It functions as a valuable prognostic indicator across various cancer types^6^.

A comprehensive literature review did not reveal any studies pertaining to the association between NPS and in-hospital mortality following TVS. This study seeks to investigate the predictive value of NPS for in-hospital mortality among patients undergoing TVS.

## METHODS

A total of 360 consecutive patients diagnosed with severe TV disease who underwent TVS at our institution between February 2019 and December 2023 were included in this retrospective study. The exclusion criteria comprised patients with systemic inflammatory disorders, chronic autoimmune diseases, active systemic infections, those necessitating immediate surgical intervention for any reason, and individuals with a documented history of types 1–3 pulmonary arterial hypertension. Following the application of these exclusion criteria, a total of 280 patients were ultimately included in the study.

Prior to the procedure, blood samples were obtained from a forearm vein following a 12-h fasting period. The analysis of blood albumin levels was performed using a Cobas 8000 c502 analyzer (Roche Holding AG, Basel, Switzerland). Furthermore, the length of the hospital stay (LoS) was documented. The EuroSCORE II was computed for each patient (https://www.euroscore.org).

All subjects underwent preprocedural transthoracic echocardiography. A qualified echocardiographer conducted all echocardiographic assessments using a GE Vingmed Ultrasound AS instrument from Horten, Norway, equipped with a 3.2 MHz adult probe.

The Naples prognostic score comprises the following variables: serum albumin and total cholesterol levels, NLR, and LMR^6^. The Naples Prognostic Score (NPS) is based on four blood markers that assess a patient's nutritional status and systemic inflammation. The criteria for evaluating NPS are as follows:

1. NLR greater than 2.96
2. LMR less than 4.44
3. Total cholesterol (TC) at 180 mg/dL or above
4. Serum albumin levels below 4 g/dL

Each parameter contributes one point to the NPS, resulting in a total score that can range from 0 to 4. In this study, patients were classified as having a low NPS if their score was 0, 1, or 2, and as having a high NPS if their score was 3, 4.

The study protocol was approved by the local ethics committee and was conducted in accordance with the Declaration of Helsinki. Informed consent was obtained from all patients.

### Statistical analyses

The baseline characteristics of the study population were divided according to NPS at admission; low (0–1–2) and high (3–4). The normality distribution of continuous variables was analyzed using analytical and visual methods. To determine the predictive value of the NPS value for the development of in-hospital mortality, receiver operating characteristic (ROC) curve was used. Effects of individual exposure were reported using hazard ratio (HR) and 95%CI. Univariate and multivariate logistic regression analyses were performed to determine the independent determinants of in-hospital mortality. The level of statistical significance was set at p<0.05, and an area under the ROC curve.

## RESULTS

The baseline demographic, clinical, and procedural characteristics of the patients are summarized in Table 1. The study sample consisted of 280 patients with severe TV disease who underwent TVS. The mean age of the sample was 59±12 years. One hundred seventy-two (61.4%) patients were female. The sample was divided into two groups: low NPS and high NPS. There was no difference between the patients with a low NPS group and those with a high NPS group in age, presence of diabetes mellitus (DM), and pacemaker or implantable cardioverter defibrillators (ICD). In the High NPS group, female gender and atrial fibrillation were less common, while smoking was more prevalent. When echocardiographic parameters were evaluated, tricuspid annular plane systolic excursion and TV diameter were similar between the groups, while pulmonary arterial systolic pressure was higher and left ventricular ejection fraction was lower in the high NPS group. When evaluating laboratory parameters, levels of hemoglobin, albumin, total cholesterol, and lymphocyte counts were higher in the low NPS group, while neutrophil and monocyte counts, as well as creatinine levels, were elevated in the high NPS group. EuroSCORE II was higher in the high NPS group compared to the low NPS group.

**Table 1:** Demographic, clinical, and procedural characteristics of the study group.

	Low NPS (n=156)		High NPS (n=124)		All (n=280)		p
Age (years)	58	±12	61	±11	59	±12	0.116
Gender, n (%) (female)	109	69.9	63	50.8	172	61.4	**0.001**
Active smokers, n (%)	10	6.5	20	16.3	30	10.8	**0.009**
Patients with DM, n (%)	57	36.8	44	35.8	101	36.3	0.863
Pacemaker or ICD, n (%)	8	5.1	7	5.6	15	5.4	0.849
Atrial fibrillation, n (%)	129	82.7	89	71.8	218	77.9	**0.029**
TAPSE (mm)	17.1	±3.6	17.4	±3.9	17.2	±3.7	0.739
PASP (mmHg)	50	±15	54	±15	52	±15	**0.023**
Tricuspid valve diameter, mm	34.02	±13.2	34.24	±14.3	34.12	±13.7	0.416
LVEF (%)	55	±8	52	±10	54	±9	**0.003**
Hemoglobin level (g/L)	12.91	±1.76	11.91	±2.25	12.47	±2.05	**<0.001**
Monocyte count (10^3^/mL)	0.57	±0.17	0.7	±0.23	0.63	±0.21	**<0.001**
Neutrophil count (10^3^/mL)	4.37	±1.3	5.57	±1.81	4.9	±1.66	**<0.001**
Lymphocyte count (10^3^/mL)	2.21	±0.66	1.52	±0.64	1.9	±0.7	**<0.001**
Creatinine level (mg/dL)	1	(0.71–1.04)	1.18	(0.74–1.2)	1.07	(0.71–1.09)	**0.001**
Albumin level (mg/dL)	4.25	±0.43	3.64	±0.65	3.98	±0.62	**<0.001**
Total cholesterol (mg/dL)	177.6	±46.6	143.9	±29.6	162.6	±43.3	**<0.001**
NLR	1.97	(1.53–2.6)	3.65	(2.96–5.13)	2.62	(1.74–3.57)	**<0.001**
LMR	3.96	(2.97–5.03)	2.11	(1.5–2.88)	3	(2.1–4.38)	**<0.001**
EuroSCORE II	2.46	(1.68–3.93)	3.46	(2.06–5.46)	2.8	(1.88–4.87)	**0.001**
LoS, (days)	11	(8–16)	13	(9–22)	12	(8–18)	**0.005**
In-hospital mortality, n (%)	8	5.1	35	28.2	43	15.4	**<0.001**

NPS: Naples prognostic score; DM: diabetes mellitus; ICD: implantable cardioverter defibrillators; TAPSE: tricuspid annular plane systolic excursion; PASP: pulmonary arterial systolic pressure; LVEF: left ventricular ejection fraction; NLR: neutrophil-lymphocyte ratio; LMR: lymphocyte-monocyte ratio; LoS: length of hospital stay. Values in bold are statistically significant at p<0.005.

### In-hospital outcomes

The study's primary outcome—in-hospital mortality—was significantly higher in patients with a high NPS group than in those with a low NPS group (35 [28.2%] vs. 8 [5.1%], p<0.001). In parallel, the length of hospital stay (LoS) was significantly higher in patients with a high NPS than in those with a low NPS (Table 1).

### Independent predictors of in-hospital mortality

Univariate logistic regression analysis revealed significant correlations between in-hospital mortality and age, hemoglobin, the presence of a pacemaker or ICD, atrial fibrillation, DM, creatinine, EuroSCORE II, and NPS (Table 2). Further analysis of these variables using the multivariate logistic regression analysis indicated that EuroSCORE II (HR 1.049, 95%CI 1–1.1; p=0.049), and NPS (HR 1.606, 95%CI 1.136–2.27; p=0.007), were independent predictors for the development of in-hospital mortality (Table 2).

**Table 2:** Results of the univariate and multivariate analyses of the variables in terms of their prognostic value in predicting in-hospital mortality in patients undergoing tricuspid valve surgery.

	Univariate analysis			Multivariate analysis		
	Univariate HR, 95%CI		p-value	Multivariate HR, 95%CI		p-value
EuroSCORE II	1.063	(1.014–1.114)	**0.011**	1.049	(1–1.1)	**0.049**
NPS	1.067	(1.147–2.25)	**0.006**	1.606	(1.136–2.27)	**0.007**

HR: hazard ratio; CI: confidence interval; p: probability statistic; NPS: Naples prognostic score. Values in bold are statistically significant at p<0.005.

A simple sampling of 1,000 samples was performed. The bootstrap results showed a EuroScore CI -0.23 to 1.68, p-value of 0.165, and an NPS CI 1.004 to 1.111, p-value of 0.04. Bootstrapping confirmed that the NPS remained significant.

**Figure 1:**
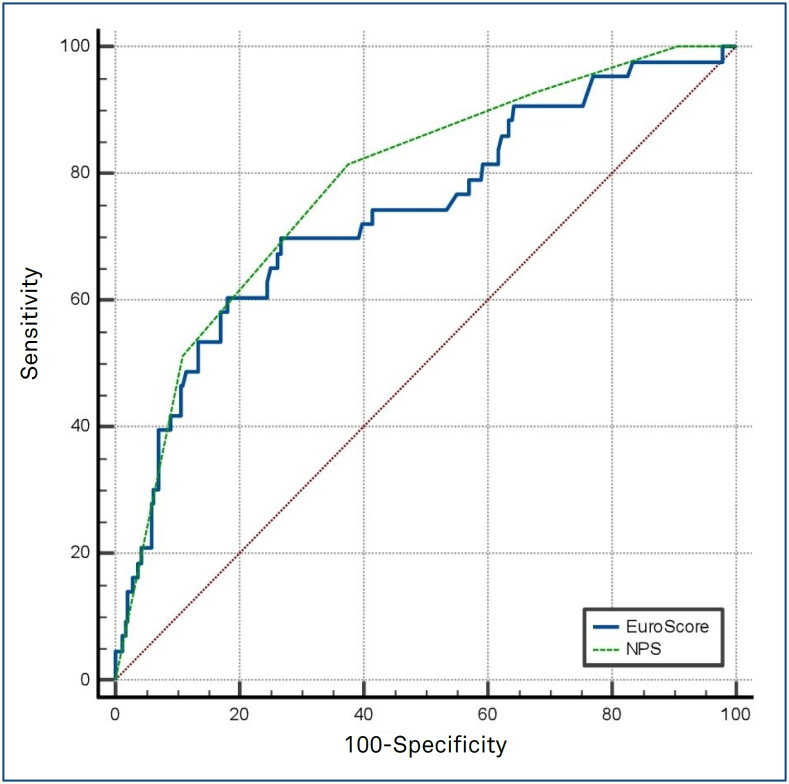
Receiver operating characteristic curve analysis of Naples prognostic score and EuroScore in patients undergoing tricuspid valve surgery.

In the ROC analysis, NPS optimal cut-off value of >2 predicted in-hospital mortality with 81.4% sensitivity and 62.5% specificity (area under the curve [AUC]: 0.782 [95%CI 0.729–0.829, p=0.003]), and EuroScore (AUC: 0.737 [95%CI 0.681–0.788, p=0.045]) (Figure 1).

## DISCUSSION

This study's findings indicate that the high NPS is an independent predictor of in-hospital mortality in patients undergoing TVS. To the best of the authors’ knowledge, this is the first study to investigate the prognostic value of the NPS in predicting in-hospital mortality for patients undergoing TVS. Our results suggest that the NPS may be valuable in assessing an individual patient's mortality risk after TVS. The NPS is relatively simple to calculate, and its components often serve as hematological and biochemical indicators. By calculating the score early, we may be able to identify patients at higher risk for mortality, allowing us to take a more cautious approach in managing their care.

Notwithstanding its widespread occurrence and links to higher mortality and morbidity, surgery for TR is rarely conducted. Consequently, only 10% of patients diagnosed with TR receive surgical intervention^7^. Unlike aortic stenosis or mitral regurgitation, TR progresses slowly, with negative outcomes often emerging after many years, contributing to its misleadingly benign reputation. However, research consistently indicates a significant in-hospital mortality rate of 8–10%^5^.

The NPS is an uncomplicated and readily accessible scoring system derived from serum albumin, total cholesterol, the NLR, and the LMR. In 2017, NPS was first utilized to assess the long-term survival of patients who underwent surgery for colorectal cancer^6^. Since then, researchers have examined the prognostic value of NPS in different cancers and chronic disease groups. A meta-analysis focusing on lung cancer patients revealed that a low NPS correlates with improved overall survival and disease-free survival rates^8^. Limited studies have explored the significance of NPS in predicting outcomes in cardiovascular diseases. Outstandingly, NPS has been shown to influence both in-hospital and midterm mortality rates, as well as rehospitalization among patients with functional class-3 and -4 heart failure resulting from decompensated heart failure^9^. In addition, Hakgor et al.'s study found that a high NPS was associated with a higher rate of in-hospital mortality following Transcatheter Aortic Valve Implantation^10^.

There exists a limited number of studies in the literature that examine the relationship between inflammation and mortality in patients undergoing TV surgery. In a study conducted by Yoon et al., preoperative systemic inflammation, as assessed by the systemic immune-inflammation index, was found to be significantly associated with an elevated risk of postoperative composite complications and all-cause mortality within 1 month following TV surgery^11^.

Although the link between hypoalbuminemia and poor surgical outcomes has been recognized for years, the underlying mechanisms remain unclear. Hypoalbuminemia, a marker of malnutrition, is associated with higher in-hospital and long-term mortality in patients before cardiac surgery^12^. This nutritional deficiency significantly heightens frailty in patients, negatively affecting their prognosis. Numerous scoring systems evaluating nutrition and physical status have been used in large-scale global studies, consistently linking functional and metabolic frailty to unfavorable outcomes^12^. Several reasons are proposed for this relationship. First, low serum albumin levels serve as an early warning sign for post-surgery outcomes, since preexisting inflammation can worsen the inflammatory response following surgery. Hypoalbuminemia often arises from chronic health conditions, with its severity reflecting the intensity of the inflammatory insult and associated mortality risk. Second, many studies link hypoalbuminemia to nutritional status, as albumin is a key indicator of in-hospital malnutrition and frailty. A low serum albumin level after surgery may indicate an unhealthy body mass index, which is connected to poor nutrition and inefficient metabolism, impairing the body's inflammatory and immune responses. Third, among elderly patients, hypoalbuminemia was common; serum albumin levels tend to decline with age, gradually decreasing from around 50 years old. Fourth, albumin's pharmacological roles as an antioxidant and transporter suggest that its deficiency might impair these functions, contributing to adverse outcomes^12^. In our study, it was observed that the in-hospital mortality rate was elevated among patients with TVS presenting lower levels of albumin, resulting in an increased NPS.

Neutrophils play a crucial role in managing inflammatory responses and releasing inflammatory mediators. When the inflammatory response is hyperactivated, it can negatively impact myocardial function due to maladaptive changes. This may result in cardiac metabolic disorders, reduced contractility, ventricular dysfunction, and myocardial remodeling^13^. In contrast, lymphocytes serve to regulate the immune system's response. Notably, in patients with chronic heart failure, chronic inflammation, oxidative stress, and neurohormonal activation elevate plasma cortisol levels and increase catecholamine release. This process downregulates lymphocyte differentiation and proliferation, further leading to lymphocyte apoptosis^14^. Previous studies indicate that a low lymphocyte count can serve as an early marker of physiological stress^15^. In the study conducted by Shahim et al., it was shown that preoperative NLR predicted short- and long-term mortality after Aortic Valve Replacement surgery in patients diagnosed with severe AS^16^. It remains uncertain whether elevated NLR directly contributes to the development of TV diseases or if it is simply a marker indicating other systemic conditions that persist after TVS and raise mortality risks in the high NLR group. It is also unknown whether elevated NLR causes the increased mortality or merely reflects underlying etiological factors. If elevated NLR is connected to the development of TV diseases, the persistent higher mortality rates following TVS in the high NLR group imply that TV disease is part of a systemic process, not confined to TVS. Alternatively, elevated NLR might be a marker or component of a broader systemic abnormality with multiple effects. TV diseases could be one manifestation among others that develop over time post-TVS, contributing to higher mortality. Monocytes, which function as the precursors to macrophages, serve as indicators of the equilibrium between inflammation and immunity. The presence of preoperative monocytosis signifies insufficient activation of macrophages, leading to an imbalance favoring inflammation over immunity, thereby contributing to a less favorable prognosis for patients undergoing cardiac surgery. The LMR more effectively integrates the clinical significance of lymphocytes and monocytes. A lower LMR (less than 3.58) is associated with an increased risk of mortality among patients undergoing on-pump cardiac surgery, as evidenced by a cohort study with a 4-year follow-up period^17^. Our study observed that NPS increased in TVS patients with high NLR and low LMR, leading to a higher in-hospital mortality rate.

Serum cholesterol levels serve as indicators of nutritional status. Total cholesterol constitutes a complex lipid profile parameter that is associated with digestive functions, reflecting chronic inflammation and daily nutritional status^18^. Consequently, it may exert a direct influence on the primary endpoint of the study.

Moreover, the EuroSCORE and STS scores have not undergone validation for isolated TV surgery, as conventional scoring systems do not take into account the factors that contribute to heightened mortality in tricuspid operations. In our research, consistent with existing literature, a higher EuroSCORE II was observed in the high NPS group that underwent TVS and experienced elevated in-hospital mortality rates.

Elevated NPS scores were associated with a heightened risk of in-hospital mortality and LoS, irrespective of the presence of right ventricle dysfunction or underlying comorbidities. Consequently, the NPS may be employed to stratify the risk of patients undergoing TVS, potentially leading to improved surgical outcomes for TV procedures in the future.

### Limitations

First, it was a retrospective, observational study conducted at a single center. Second, we only assessed baseline albumin, cholesterol, and hematological values; tracking changes through serial measurements could provide additional prognostic value. Additionally, we did not compare the NPS with traditional risk scoring systems, such as the TRI-SCORE, which is a new risk score designed to predict in-hospital mortality after isolated TV surgery^5^. Furthermore, relying solely on ROC analysis to identify the optimal NPS cut-off is a methodological limitation of the study.

## CONCLUSION

This study represents the first comprehensive examination of the utility of the NPS as a preoperative risk assessment tool for patients undergoing TVS. This study's findings show that the NPS independently predicts in-hospital mortality for patients undergoing TVS.

## ETHICAL APPROVAL

The study protocol was approved by the local ethics committee and was conducted in accordance with the Declaration of Helsinki. Informed consent was obtained from all patients.

## Data Availability

The datasets generated and/or analyzed during the current study are available from the corresponding author upon reasonable request.
